# Characteristics of Frailty in Perimenopausal Women with Long COVID-19

**DOI:** 10.3390/healthcare11101468

**Published:** 2023-05-18

**Authors:** Alba Navas-Otero, Andrés Calvache-Mateo, Javier Martín-Núñez, Irene Calles-Plata, Araceli Ortiz-Rubio, Marie Carmen Valenza, Laura López López

**Affiliations:** Physical Therapy Department, Faculty of Health Sciences, University of Granada, 18016 Granada, Spain

**Keywords:** disability, frailty, long COVID-19 syndrome, perimenopausal women

## Abstract

The aim of this study was to compare the prevalence of risk factors for frailty between perimenopausal women with long COVID-19 syndrome, women having successfully recovered from COVID-19, and controls from the community. Women with a diagnosis of long COVID-19 and at least one symptom related to the perimenopausal period, women who had successfully recovered from COVID-19, and healthy women of comparable age were included in this study. Symptom severity and functional disability were assessed with the COVID-19 Yorkshire Rehabilitation Scale, and the presence of frailty was evaluated considering the Fried criteria. A total of 195 women were included in the study, distributed over the three groups. The long COVID-19 group showed a higher prevalence of perimenopausal symptoms and impact of COVID-19. Statistically significant differences were found between the long COVID-19 group and the other two groups for the frailty variables. When studying the associations between frailty variables and COVID-19 symptom impact, significant positive correlations were found. Perimenopausal women with long COVID-19 syndrome present more frailty-related factors and experience a higher range of debilitating ongoing symptoms. A significant relationship is shown to exist between long COVID-19 syndrome-related disability and symptoms and frailty variables, resulting in an increased chance of presenting disability.

## 1. Introduction

Coronavirus disease 2019 (COVID-19) is caused by severe acute respiratory coronavirus-2 (SARS-CoV-2) [1]. This novel coronavirus is a single-stranded ribonucleic acid (RNA) virus, primarily affecting the respiratory system [2]. The virus is transmitted from person to person via liquid droplets (sneeze, hand to mouth/eye contact, and contaminated surface or hands) [3]. It has a wide range of presentation.

At the beginning of the COVID-19 pandemic, global efforts were mostly directed to improving patient survival and equitable access to proper healthcare. As the pandemic progressed, management strategies advanced, and hospital mortality rates gradually decreased in many countries [4]. Consequently, the pandemic entered a new phase, at the end of 2021, 23 months after the first reported case of COVID-19, and the global number of infections was complex. The estimated data report around 260 million cases and 5.2 million deaths worldwide. The registered cases unfortunately continued increasing every day due to the emergence of new variants of the virus. Spain is considered one of the two most affected nations in Europe alongside Italy [5]. Although it was crucial to prioritize minimizing the immediate impact, there is great concern regarding the enduring repercussions of the pandemic [6].

Long COVID-19 syndrome has emerged as a new clinical condition from the COVID-19 situation, with an estimated general prevalence of 10 to 35% and significant differences between hospitalized (85%) and non-hospitalized (34.8%) populations [7,8,9]. According to National Institute for Health and Care Excellence (NICE) guidelines [10], long COVID-19 syndrome is defined as a set of signs and symptoms that appear during or after an initial acute infection with COVID-19. The mentioned clinical manifestations are present for more than 12 weeks and not explained by an alternative affection. Commonly, it manifests clusters of symptoms that frequently overlap, and the possibility of fluctuations and changes over time, affecting any system in the body, also exists.

The causes of this syndrome are still uncertain, and its presentation is diverse, complex, and heterogeneous with a broad range of symptoms. Predominant symptoms are fatigue, weakness, dyspnea, musculoskeletal pain, neurocognitive difficulties, and emotional disturbances [11,12,13]. These COVID-19 symptoms increase disability, negatively impact physical function and quality of life, and affect participation in general life activities and the ability to work. However, some host factors such as gender and age have been related to a higher susceptibility to developing this syndrome [14]. At least 65 million individuals around the world have long COVID-19, based on a conservative estimate incidence of 10% of infected people and more than 651 million documented COVID-19 cases worldwide [15]. The number is likely much higher due to many undocumented cases. The incidence is estimated at 10–30% non-hospitalized cases, 50–70% hospitalized cases, and 10–12% vaccinated cases.

While clinical characteristics suggest that susceptibility to COVID-19 infection is similar between men and women, clinical outcomes show that men experience both a higher severity and fatality of COVID-19 infection. In addition to differences reported in clinical presentation and outcomes [16], women seem to be more vulnerable to developing long COVID-19 symptoms. Female sex is a potential risk factor associated with the presence of symptomatology related to long COVID-19 syndrome [17]. In their study, Fernández de las Peñas et al. also describe the higher presence in women of reported long COVID-19 syndrome symptoms such as fatigue, pain symptoms, anxiety, depression, and poor sleep quality [18]. As mentioned, these disparities can be attributed to sex-based difference in immunological responses, hormonal differences, and gendered differences in behaviors such as smoking as well as the prevalence of comorbidities. Recent evidence shows that the prevalence of symptomatology is higher in people aged around 50 years old compared to the general population [13].

Long COVID-19 syndrome may be understood as a condition that often impacts daily functioning, the ability to work, social participation, exercise capacity, quality of life, and disability [19,20] in all areas of function but particularly as physical, emotional, or cognitive problems. Increased disability has an inequitable impact, both in the number of patients and in the severity of functional limitations, and is associated with increased caregiver burden, unemployment, psychological problems, and mortality [21,22,23]. In addition, long COVID-19 is associated with greater expenditure across all types of medical services, including hospitalizations, physician visits, dispensed medications, and rehabilitation [24].

Because of the residual functional disability, the cumulative healthcare costs per patient per year are comparable to those of elderly patients with severe chronic diseases. Overall, these long-term symptoms and functional limitations represent a complex interplay of multiple factors that are compatible with the existence of a situation of fragility.

Frailty has been described as a physiological state in which there is a greater vulnerability to stress factors caused by the decrease in physiological reserves [25] that, together with disease or age, affects multiple systems and is cumulative [26]. The presence of frailty implies a degree of vulnerability that necessitates complex medical care demands, and frail adults are exposed to a higher risk of poor health outcomes, including dependence and disability, considering the scarce resources of healthcare systems. However, based on the scientific evidence currently available, no previous studies have explored the risk factors for developing frailty in long COVID-19 syndrome. Specifically, the objective of this study was to compare the prevalence of frailty-related factors between perimenopausal women with long COVID-19 syndrome, women who had successfully recovered from COVID-19 (non-long COVID-19), and controls from the community.

## 2. Materials and Methods

### 2.1. Study Design and Participants

The study was a descriptive study that followed the Strengthening the Reporting of Observational Studies in Epidemiology (STROBE) guidelines [27]. Approval for the study was obtained through authorization from the Human Research Ethics Committee with registration number 2392/CEIH/2021. All procedures were conducted following the 2013 Declaration of Helsinki [28] and in accordance with the 1948 Universal Declaration of Human Rights [29].

Women candidates for participation were recruited from the Long-COVID Regional Association after being informed of the objective of the study. All the recruited women provided a medical diagnosis of long COVID-19 according to NICE guidelines [30,31]. During the recruitment, each subject completed and signed an informed consent statement prior to the beginning of the study.

Women who were eligible to participate were adults (≥18 years) with a medical diagnosis of long COVID-19 and actual presence of symptoms plus at least one reported symptom related to the perimenopausal period (e.g., hot flashes, depressed mood, sleep disruption). The women with long COVID-19 syndrome who agreed to participate in this study were matched by age with women who had successfully recovered from COVID-19 and healthy controls groups. The successfully recovered and control participants were recruited from the community. The inclusion criteria for the successfully recovered COVID-19 group were women who met the set diagnostic standard of testing negative at least twice in nucleic acid tests and were able to complete the test independently. The nucleic acid tests are commonly used for the diagnosis of COVID-19 through reverse transcriptase polymerase chain reaction [32]. The exclusion criteria for the three groups in this study were presenting cognitive, vascular, neurological, or orthopedic disorders that could limit the performance of the test. They signed an informed consent form after being informed about the study conditions [33].

### 2.2. Procedures and Measures

At the beginning of the study, evaluation was performed by one therapist through an interview. This interview included an evaluation of demographic and clinical features, reported perimenopausal symptoms [33], comorbidities [34], the impact of COVID-19 [35], and the presence of frailty.

The evaluation of demographic and clinical features included age, body mass index, and time since infection. Perimenopausal symptoms were assessed according to the description of the most prevalent symptoms during the perimenopause reported by Woods et al. [33]. The presence of comorbidities was evaluated using the Charlson Comorbidity Index (CCI) [34]. It provides a weighted score of comorbidities using 19 items. Higher scores indicate a more severe condition. The CCI has been used extensively in clinical research to address the cofounding influence of comorbidities. It has also been used in clinical research to address the effects of comorbidities, predict outcomes, and establish a standardized guide of comorbidities abstracted from medical records or administrative databases as well as for self-reported comorbidities. According to this test, a score of 0 to 1 is considered as no comorbidity, low comorbidity scores up to 2 points, and high comorbidity is more than 3 points. The impact of COVID-19 was evaluated with the COVID-19 Yorkshire Rehabilitation Scale (C19-YRS) [35]. This instrument assesses participants’ clinical profile in the last 7 days, including symptom severity and functional disability through the activities and participation domains of the International Classification of Functioning, Disability, and Health. The C19-YRS consists of 22 items with each item rated on an 11-point numerical scale from 0 (none of this symptom) to 10 (extremely severe level or impact). It is divided into three subscales (range of total scores for each subscale): symptom severity (0–100), functional disability (0–50), and additional symptoms (0–60). Additionally, overall health is measured from 0 to 10, where lower scores indicate poorer overall health. The content validity of the C19-YRS has been demonstrated in previous studies, and it is used in several rehabilitation services. Good data quality, good scaling, targeting, and good reliability of individual subscales have also been shown [35,36].

The presence of frailty was evaluated using the Fried criteria. Specifically, body mass index, self-reported exhaustion, weakness, gait speed, and physical activity level were recorded [37]. Despite there still being no gold standard for measuring frailty, other authors have used these five components for addressing the presence of frailty [38].

The body mass index of all participants was calculated after being measured by research staff and used as a reference value for the Fried criteria for assessing frailty-related low weight [39]. According to the World Health Organization (WHO), body mass index (BMI) is defined as a simple indicator of the relationship between weight and height. Self-reported exhaustion during the last 7 days was assessed with the fatigue subscale of the PROMIS-29 (Patient-Reported Outcomes Measurement Information System-29) tool [40]. The fatigue subscale of the PROMIS-29 is composed of 4 items that are scored from 1 to 5 where a higher score indicates higher levels of fatigue. This measurement has undergone extensive development, study, and application in a variety of diseases [41]. Sufficient structural validity, internal consistency, and measurement invariance has been found in populations with and without chronic conditions [42]. Weakness was evaluated with the Five Sit-to-Stand Test (5STS) [43]. This test consists of sitting down and getting up five times from a chair as fast as possible. It is the most widely used method to measure muscle performance [44] and also reflects endurance, balance, and mobility [45]. This test has demonstrated good predictive value for detecting frailty [46]. Gait speed was assessed over a distance of 2.4 m [47]. This test consists of the person walking at his/her usual walking pace “as if he/she were walking down the street to go shopping”. Any assistive devices (e.g., cane, crutch) can be used. Two measurements are taken and the higher is used. Gait speed test is a fast, inexpensive, and easy measure of physical capacity with documented predictive value and validity for major clinical outcomes in our population [48].

Level of physical activity was assessed using the International Physical Activity Questionnaire (IPAQ) [49]. This tool consists of 7 items that provide information about the time a person spent in moderate and vigorous intensity activities, walking, and sitting during the last 7 days. It provides information about METs (resting metabolic units), where a higher number of METs indicates a higher level of physical activity. The IPAQ is one of the best and most widely used tools to evaluate physical activity [50]. This instrument has an appropriate validity and reliability for the measurement of physical activity and good reliability coefficients for application in a Spanish population [51].

### 2.3. Statistical Analysis

Normality testing of variables was performed using the Kolmogorov–Smirnov test. Categorical data are presented as frequencies and percentages and continuous variables are presented as mean (standard deviation; SD) or median (interquartile range; IQR), depending on normality. The chi-squared test was used to examine relationships between categorical variables. For group comparisons, an ANOVA (ANalysis Of VAriance) or the Kruskal–Wallis test was used, including post-hoc Mann–Whitney U tests. A value of *p* < 0.05 was considered to be statistically significant.

Assuming normal distribution of estimated proportions for the primary descriptive, cross-sectional analysis (e.g., proportion of participants with frailty), a sample size of 68 participants per group would have guaranteed a maximum margin of error (confidence interval) of ±10% with α = 0.05 and was ultimately considered an acceptable compromise between the feasibility and generalizability of the study results.

Bivariate correlation analysis was conducted between the Yorkshire Rehabilitation Scale subscores and the frailty variables in the long COVID-19 group. Spearman’s rho correlation coefficient was used to determine the association. The strength of the correlations was based on the criteria described by Cohen [52], who has written extensively on the subject and described the magnitude of correlations as follows: >0.5 is large, 0.5–0.3 is moderate, 0.3–0.1 is small, and anything <0.1 is insubstantial or trivial. A *p* value of <0.05 was considered statistically significant.

## 3. Results

**Figure 1:**
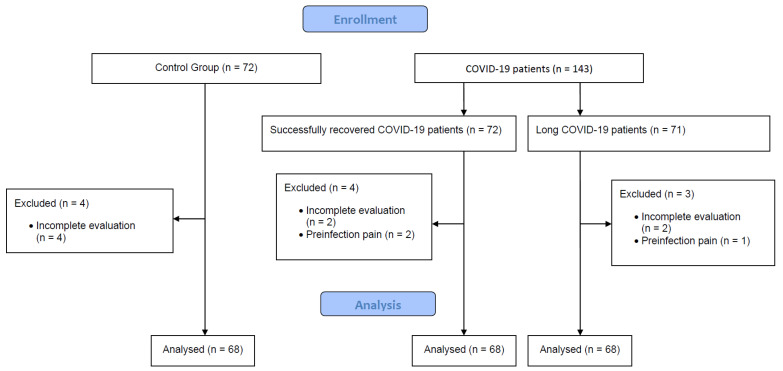
Patient selection flowchart.

A flowchart showing final patient selection is shown in Figure 1.

Table 1 shows the baseline characteristics of the women included in this study. A total of 195 women were included in the study. Of these, 68 participants were randomized into the long COVID-19 group, another 68 were recruited to the successfully recovered group, and 68 healthy women were included in the healthy control group. The estimated average time since initial infection was more than one year for both the long COVID-19 and the successfully recovered groups.

The most prevalent perimenopausal symptom reported in all groups was the presence of a depressed mood, and this symptom was very frequent in the long COVID-19 group (*p* = 0.003). The long COVID-19 group was more likely to present hot flashes, a depressed mood, and sleep disruption when compared with the healthy controls and participants in the successfully recovered COVID-19 group. The presence of comorbidities, measured with the Charlson Comorbidity Index, showed no significant differences between the groups (*p* > 0.05).

The total score for the impact of COVID-19 was higher in the long COVID-19 group in comparison with the other two groups. Additionally, higher total scores for all subscales were found in the long COVID-19 group. The greatest difference was observed in the symptom severity subscale.

The women’s scores for the different frailty variables are presented in Table 2.

The long COVID-19 group had higher self-reported exhaustion (11.82 ± 5.03, *p* < 0.05) when compared to the other groups. Comparing weakness and gait speed between groups, statistically significant differences were found between the long COVID-19 group and the other two groups (*p* < 0.05). Similar results were observed in comparing physical activity levels a, b, and c (1037.47 ± 960.78, 4732.45 ± 3592.17, 7455.03 ± 4736.34, respectively). No significant differences between body mass indexes were found.

The associations between frailty variables and COVID-19 symptom impact, evaluated using the Yorkshire Rehabilitation Scale, are shown in Table 3.

Significant positive correlations between Yorkshire Rehabilitation Screening subscores and frailty variables were found in the long COVID-19 group for self-reported exhaustion with symptom severity, functional disability, and additional symptoms.

The highest intensity of correlation was found between self-reported exhaustion and functional disability (r = 0.630 **, *p* < 0.001). However, no significant associations between gait speed and additional symptoms (r = −0.248, *p* = 0.074) or between physical activity and functional disability (r = −0.229, *p* < 0.001) were found.

## 4. Discussion

The objective of this study was to compare the prevalence of frailty-related factors between perimenopausal women with and without long COVID-19 syndrome. Although there are previous studies that have explored frailty in perimenopausal women, to our knowledge this is the first study exploring frailty-related factors in perimenopausal women with and without long COVID-19 syndrome. Our obtained results are in line with those reported by Fernández de las Peñas et al. [18], suggesting that there is an additional sex-related hormonal effect in symptomatic severity for long COVID survivors.

Our results show that the prevalence of frailty-related factors in perimenopausal women with long COVID-19 syndrome could be clinically underestimated. In fact, almost all frailty-related factors were more frequent in women survivors of SARS-CoV-2 infection, specifically in those who had long COVID-19 syndrome, when compared with women who had successfully recovered from COVID-19 and healthy perimenopausal women. These frailty-related factors seem to have been undetected in women with long COVID-19 syndrome, considering the added relationship with the different COVID-19 sequelae for perimenopausal women in particular. These results are certainly relevant; according to the literature, frailty is strongly correlated to an increased risk of disability, cognitive decline, pain, falls, and death, placing individuals at higher risk of adverse outcomes and dependence in activities of daily living [53].

One of the findings of this study concerns the frailty factors in long COVID-19 syndrome in a perimenopausal sample. These results could be partially explained by the higher vulnerability related to gender (women) and age for developing long COVID-19 symptoms [18,54,55,56,57]. Different studies have reported that men tend to develop more serious cases than women, according to the clinical classification of severity. However, referring to long-term symptomatology, a study has shown that females are twice as likely to report fatigue than men of the same age, months after initial infection. Another study, from Munblit et al., found that female sex is associated with persistent symptoms [58]. Zhang et al. report that female sex is also associated with a higher risk of post-COVID-19 fatigue [58]. Therefore, gender is a risk factor that causes higher levels of severity and mortality in patients with COVID-19.

These factors may also account for the fact that, as expected, middle-aged women generally have a higher risk of experiencing a range of debilitating ongoing symptoms, such as fatigue, weakness, breathlessness, muscle pain, anxiety, depression, and “brain fog” [59].

Furthermore, our study found a significant relationship between long COVID-19-related disability and self-reported exhaustion, weakness, and physical activity in perimenopausal women. It also found a significant relationship between gait speed and all COVID-19-related disability subscores, except for additional symptoms. In agreement with our results, a recent study provided evidence about the relationship between comorbidities and etiologic frailty, with disability as a consequence [37]. Similarly, a study by Lindahl et al. demonstrated that women report more symptoms and lower quality of life than men with a long COVID-19 diagnosis [60].

Some previous studies have reported the most common long COVID-19 symptoms as tiredness, fatigue, sleep problems, and dyspnea, among others [59,61]. These symptoms are usually associated with frailty in different populations, such as the elderly. However, our study is the first to explore them in a perimenopausal population with coexisting long COVID-19 syndrome.

In addition, the results of the present study show differences in self-reported exhaustion per group, which was higher in the women with long COVID-19. In this regard, other authors have suggested fatigue as a dominant symptom, along with other features reminiscent of the acute infection [62].

Our findings also report unequal results in relation to muscle weakness and gait speed, echoing the finding by Dinglas et al. [63] that they are key contributors to the persistent disability of women with long COVID-19. As such, muscle weakness, deconditioning, myopathies, and neuropathies may be crucial secondary results of COVID-19, potentially leading to increased morbidity, disability, and mortality in the long-term [64].

We found that physical performance was lower in women with long COVID-19 than in those who had successfully recovered from COVID-19 and healthy controls. This observation is not surprising when considering the association between muscle weakness, poor physical performance, and gait speed, with the latter being higher in women with long COVID-19. Beyond persistent symptoms, patients with long COVID-19 may present several clinical findings as a consequence of this condition [65].

### 4.1. Clinical Implications

- Perimenopausal women with long COVID-19 syndrome present frailty-related factors and also suffer a higher range of debilitating ongoing symptoms than successfully recovered and healthy women.
- A rehabilitation program for perimenopausal women with long COVID-19 syndrome is needed.
- The rehabilitation program should focus on frailty symptoms.

### 4.2. Strengths and Limitations of the Study

There are some limitations that should be mentioned. First, all Fried criteria [37] should have been used to characterize frailty in our participants, as they provide a standardized definition of frailty. However, a review concluded that the most common identifying factors for frailty are physical functioning and gait speed [66], as included in our study. The choice of criteria to be included in the definition of frailty is a highly controversial issue with important implications. Second, we identified perimenopausal women based on anamnestic data collected through personal interviews. This can be interpreted as a limitation due to the existence of specific questionnaires, but other studies have used the same reported symptoms to identify perimenopausal populations [67,68,69]. These possible biases in self-reported symptoms may have affected our results, and so our conclusions are not generalizable to other populations.

Third, the design of our study made it possible to measure the prevalence of frailty aspects, but longitudinal studies assessing post-diagnostic changes in frailty measures are needed to better understand the impact of long COVID-19 on frailty. Finally, the weight-loss criterion was not considered in the evaluation of frailty-related factors due to the scarcity of this data in our sample. This may have influenced our evaluation of relationships between COVID-19-related sequelae and the presence of frailty-related factors. Although some studies have described other findings in relation to long COVID-19, there is an urgent need for research into the clinical implications [70]. 

## 5. Conclusions

Perimenopausal women with long COVID-19 syndrome present more frequent frailty-related factors and also experience a higher range of debilitating ongoing symptoms than the general population. Moreover, a significant relationship has been shown to exist between long COVID-19 syndrome-related disability and symptoms on one hand and between self-reported exhaustion, weakness, and gait speed on the other, resulting in an increased chance of disability.

## Figures and Tables

**Table 1:** Anthropometric characteristics by group.

	Long COVID-19 Group (n = 68)	Successfully Recovered COVID-19 Group (n = 68)	Healthy Control Group (n = 68)	F
Demographics and clinical features				
Age (years)	42.62 ± 9.65	44.58 ± 16.12	43.62 ± 19.92	1.362
Body mass index (kg/cm^2^)	25.98 ± 5.96	25.18 ± 4.11	25.53 ± 4.14	0.304
Time since infection (months)	16.24 ± 4.9	18.07 ± 6.5	-	-
Perimenopausal symptoms (%)				
Hot flashes	13.8 (9)	10.7 (7)	12.3 (8)	0.351
Depressed mood	46.1 (30)	30.7 (20)	24.6 (16)	0.003
Sleep disruption	32.3 (21)	21.5 (14)	16.9 (11)	0.012
Presence of comorbidity	1.58 ± 0.63	1.62 ± 0.84	1.48 ± 0.71	32.527
Impact of COVID-19				
Symptom severity	42.7 ± 0.36	2.7 ± 11.8	-	-
Functional disability	20.8 ± 15.2	5.1 ± 3.7	-	-
Additional symptoms	19.6 ± 12.8	6.4 ± 2.5	-	-
Overall health	4.6 ± 2.1	1.7 ± 1.4	-	-

Note: Results are shown as mean ± standard deviation.

**Table 2:** Frailty variables per group.

	Long COVID-19 Group (n = 68)	Successfully Recovered COVID-19 Group (n = 68)	Healthy Control Group (n = 68)	F	Global ANOVA *p* Value	Post-Hoc Pairwise Test
Body mass index (kg/cm^2^)	25.98 ± 5.96	25.18 ± 4.11	25.53 ± 4.14	0.304	0.738	a = 0.983 b = 0.902 c = 0.899
Self-reported exhaustion	11.82 ± 5.03	14.23 ± 3.58	15.93 ± 3.05	9.52	*p* < 0.001	a = 0.023 b < 0.001 c < 0.226
Weakness	14.23 ± 5.98	8.01 ± 2.58	7.73 ± 2.86	28.091	*p* < 0.001	a < 0.001 b < 0.001 c = 0.789
Gait Speed (m/s)	1.84 ± 0.74	1.96 ± 0.52	3.85 ± 1.84	31.737	*p* < 0.001	a < 0.001 b < 0.001 c = 0.623
Physical activity (METs)	1037.47 ± 960.78	4732.45 ± 3592.17	7455.03 ± 4736.34	38.754	*p* < 0.001	a < 0.001 b = 0.002 c < 0.001

Note: Results are shown as mean ± standard deviation. a: comparison between the long COVID-19 group and the successfully recovered COVID-19 group (*p* < 0.05) b: comparison between the long COVID-19 group and the healthy control group (*p* < 0.05) c: comparison between the successfully recovered COVID-19 group and the healthy control group (*p* < 0.05).

**Table 3:** Bivariate associations between Yorkshire Rehabilitation Scale subscores and frailty variables in the long COVID-19 group.

	Symptom Severity	Functional Disability	Additional Symptoms	Overall Health
Body mass index	−0.072	0.083	−0.001	−0.064
Self-reported exhaustion	0.465 **	0.630 **	0.199 *	−0.211 *
Weakness	0.498 **	0.627 **	0.230 **	−0.579 **
Gait Speed	−0.440 **	−0.529 **	−0.248	0.411 *
Physical activity	−0.189 **	−0.229 **	−0.450 *	0.592 **

Note: Results are shown as correlation coefficient (r) * *p* < 0.05, ** *p* > 0.001.

## Data Availability

No additional data are available.
